# A systematic review of the effectiveness and cost-effectiveness of peer education and peer support in prisons

**DOI:** 10.1186/s12889-015-1584-x

**Published:** 2015-03-25

**Authors:** Anne-Marie Bagnall, Jane South, Claire Hulme, James Woodall, Karen Vinall-Collier, Gary Raine, Karina Kinsella, Rachael Dixey, Linda Harris, Nat MJ Wright

**Affiliations:** Centre for Health Promotion Research, Leeds Beckett University, Leeds, LS1 3HE UK; Academic Unit of Health Economics, Leeds Institute of Health Sciences, University of Leeds, LEEDS, LS2 9LJ UK; Spectrum Community Health CIC, White Rose House, West Parade, Wakefield, WF1 1LT UK; Leeds Community Healthcare HMP Leeds Healthcare Department, 2 Gloucester Terrace, Armley, Leeds, LS12 2TJ UK

**Keywords:** Systematic review, Prisoners, Prisons, Peer interventions, Peer education, Peer support, Health promotion, Health inequalities

## Abstract

**Background:**

Prisoners experience significantly worse health than the general population. This review examines the effectiveness and cost-effectiveness of peer interventions in prison settings.

**Methods:**

A mixed methods systematic review of effectiveness and cost-effectiveness studies, including qualitative and quantitative synthesis was conducted. In addition to grey literature identified and searches of websites, nineteen electronic databases were searched from 1985 to 2012.

Study selection criteria were:

Population: Prisoners resident in adult prisons and children resident in Young Offender Institutions (YOIs).

Intervention: Peer-based interventions.

Comparators: Review questions 3 and 4 compared peer and professionally led approaches.

Outcomes: Prisoner health or determinants of health; organisational/process outcomes; views of prison populations.

Study designs: Quantitative, qualitative and mixed method evaluations.

**Results:**

Fifty-seven studies were included in the effectiveness review and one study in the cost-effectiveness review; most were of poor methodological quality. Evidence suggested that peer education interventions are effective at reducing risky behaviours, and that peer support services are acceptable within the prison environment and have a positive effect on recipients, practically or emotionally. Consistent evidence from many, predominantly qualitative, studies, suggested that being a peer deliverer was associated with positive effects. There was little evidence on cost-effectiveness of peer-based interventions.

**Conclusions:**

There is consistent evidence from a large number of studies that being a peer worker is associated with positive health; peer support services are also an acceptable source of help within the prison environment and can have a positive effect on recipients. Research into cost-effectiveness is sparse.

**Systematic review registration:**

PROSPERO ref: CRD42012002349.

## Background

Offender health is a priority for the Department of Health in England and Wales [1] because ill health is more prevalent in prisoners than the general population [2], and prisoners experience significant health inequalities associated with multi-faceted social problems. [3,4] In December 2013, the prison population of England and Wales was 85,429 [5] - high by European standards [6] - with a relative increase in prisoners aged over 50 years [7]. The global prison population has also grown exponentially in all five continents, to a reported 10 million [8]. Imprisonment can produce adverse health impacts, particularly in mental health [9,10]; in 2012, for example, there were 23,158 self-harm incidents in prisons, affecting approximately 6,761 individuals. Younger prisoners, female prisoners and prisoners in the early stages of custody were most likely to self-harm. Suicides are reported to be 102.6 per 100,000 prisoners, compared with 10–12 per 100,000 in the general population [11]. Evidence shows that prisoners engage in riskier health behaviours, such as drug and alcohol misuse [4] and smoking [12]. Inequalities in long term conditions also exist; with over a quarter of newly sentenced prisoners reporting a long-standing physical disorder or disability [13]. Evidence suggests that women prisoners [13,14] and older prisoners [7] have greater physical health needs.

Since 2006, the NHS has had responsibility for prison healthcare in England and Wales, with a duty to provide services equivalent to those in the community and, since April 1^st^ 2013, NHS England took responsibility and oversight for commissioning all health services (with the exception of some emergency care, ambulance services, out of hours and 111 services) for people in prisons in England through ‘Health and Justice’ commissioning teams. [15] While many offenders experience barriers accessing health services outside of prison [16], prison health services can potentially improve prisoners’ physical and mental health [17]. NHS England have a clear remit for commissioning health promotion in prison, supported by the Ministry of Justice who are responsible for wider health promotion through non-clinical service provision, such as exercise promotion delivered by qualified prison gym staff [15].

Peer-based interventions, where prisoners provide education, support or advice to other prisoners, can contribute to achieving health and social goals within the prison environment and beyond [18]. A 2002 survey estimated that seven percent of prisoners played peer support roles [19]. Justifications include: ability of peers to connect with other prisoners [20] and to have social influence with vulnerable populations resistant to professional advice [21,22]; direct benefits for the peer deliverers themselves [20,23]; wider benefits for the prison system including effective use of resources [24,25]; expanding the range of health services in the criminal justice system [26].

There is evidence of peer interventions operating across prisons globally, ranging from HIV/AIDS programmes in Mozambique and Siberia [27,28] to peer-led emotional support schemes in Israeli prisons [29]. Nevertheless, recent commentators have argued that the emphasis placed on health promotion intervention varies significantly across the world’s prison systems. The WHO’s health-promoting prison philosophy, for example, is less well developed in resource-poor regions, like sub-Saharan Africa [30]. This is reiterated in recent reviews which have shown that most published accounts of peer interventions come from prison systems in the UK, US, Canada and Australia [31].

Peer support is an established feature of prison life in England and Wales, for example the Listeners scheme, developed by the Samaritans and first launched in 1991 at HMP Swansea [32] as part of a suicide prevention strategy, now operates across most prisons in England and Wales. Other peer–based interventions in English and Welsh prisons address substance misuse, violence reduction, support for new prisoners, translation services, housing and employment advice and mentoring schemes [23] and, more recently, health trainer schemes [26].

There is an extensive evidence base on peer roles for improving access to healthcare services and removing barriers to health in the general population [33,34], but more needs to be known about the effectiveness of these interventions in prison settings, especially given their prominence [19,23,35]. The international literature on effectiveness of different types of prison-based peer education and support has not been systematically reviewed. One literature review of prison-based peer education schemes noted the dearth of evidence demonstrating effectiveness, despite positive impacts reported by some studies [22], while a recent systematic review of peer health promotion concluded that peer education could impact positively on attitudes, knowledge and behaviours of sexual health and HIV prevention, but there was little research on other health issues [31]. Studies of peer support for suicide prevention/self-harm [20,24] report benefits of peer-delivered emotional support, such as decreased prevalence of suicide [36,37]. Peer-based interventions might be more cost-effective than professionally-delivered ones [22]. The cost-effectiveness of peer interventions promoting behavioural change has been assessed in a variety of settings and populations with mixed results [38-40], but to date there has been no systematic review of the cost-effectiveness of peer interventions on health in prison settings. This study thus addresses a knowledge gap by synthesising evidence on a range of peer-based interventions in prison settings and their effectiveness and cost-effectiveness [41].

## Methods

### Objectives

The study used standard systematic review methodology to appraise evidence on effectiveness and cost-effectiveness [42-44] with input from experts in the field, in the form of steering and advisory groups. A full study protocol was developed and peer-reviewed by the study Steering and Advisory Groups prior to publication on PROSPERO (ref: CRD42012002349 http://www.crd.york.ac.uk/prospero/display_record.asp?ID=CRD42012002349).

The main research question was:

What is the effectiveness and cost effectiveness of peer-based interventions to maintain and improve health in prisons and young offender institutions (YOIs)?

This led to four review questions:

1. What are the effects of peer-based interventions on prisoner health and the determinants of prisoner health?
2. What are the positive and negative impacts of delivering peer-based interventions on health services within prison settings?
3. How do the effects of peer-based approaches compare to those of professionally-led approaches?
4. What is the cost and cost effectiveness of peer-based interventions in prison settings?

This paper reports the findings for review questions 1, 3 and 4; review question 2 will be explored in a separate paper.

### Data sources

Sources searched for papers published from 1985 to 2012, with no language restrictions: MEDLINE; PsycINFO; CINAHL; EMBASE: International Bibliography of the Social Sciences (IBSS); ASSIA; Web of Science, Social Science Citation Index; National Criminal Justice Reference Service Abstracts; Social Services Abstracts; Sociological Abstracts; DARE; TRoPHI; DoPHER; Health Evidence Canada; ORB Social Policy Database; Social Care Online; Academic Search Complete; Cochrane and Campbell Collaboration Databases. Electronic contents lists of key journals (Journal of Correctional Health Care, Health Education & Behavior, Criminal Justice and Behavior) were also searched.

Search terms drew on results from a previous systematic scoping review on lay roles in public health [45], with further search terms identified in consultation with the project steering group.

Additional databases for the cost-effectiveness review were NHS EED and REPEC (IDEAS). Other databases were searched using an adaptation of the economics search filters developed by the NHS Centre for Reviews and Dissemination combined with the search terms used in the effectiveness literature search strategy.

Search strategies are available from the authors on request.

Unpublished (grey) literature was identified from contacts with experts, conference and dissertation abstracts, reference lists of key papers, hand searches of relevant book chapters, and searches of relevant websites. Contacts made with national and international experts included: Offender Health Research Networks (OHRNs); Prison and Offender Research in Social Care and Health (PORSCH); Samaritans (Listeners scheme); Volunteering England; National Offender Management Service (NOMS); PCTs (health trainers); Prison Officers’ Association (POA); Action for Prisoners Families; CLINKS; Prison Governors’ Association.

Practitioners and academics with expertise were contacted through academic and practice mailing lists.

### Study selection

Two reviewers independently selected studies for inclusion. Any disagreements were resolved by discussion, and a third reviewer if necessary.

### Eligibility criteria

**Population**: Prisoners resident in prisons and children in YOIs in any country, all ages, male and female.

**Intervention:** Any peer-based intervention, including peer education, peer support, peer mentoring, befriending, peer counselling and self-help groups, operating within prisons and YOIs in any country. ‘Peer’ includes prisoners and ex-prisoners delivering interventions to serving prisoners.

**Comparators:** For Review Questions 3 and 4, studies comparing peer and professionally-led approaches to the same health or social problem. For all other questions, studies with any or no comparator (or usual care).

**Outcomes:** Studies reporting any effects of peer-based interventions on prisoner health or determinants of health within the prison setting. For review question 4, papers reporting resource use/cost and/or outcome comparisons of peer-based interventions with standard care.

**Study designs:** Quantitative, qualitative and mixed method evaluations.

### Data extraction

Data were extracted onto piloted electronic forms by one reviewer and checked for accuracy by a second, with reference to a third reviewer if necessary. Data extraction fields included: Bibliographic detail; Population details; Setting/institution details; Intervention details; health or social issue; method of delivery; Outcomes.

Additional data extracted from cost-effectiveness studies were: type of economic evaluation; the basis of costing; source of cost data; cost year and discounting; summary of effectiveness and costs; cost-effectiveness/utility; sensitivity analysis and conclusions as reported by the authors.

Detailed extraction of quantitative data took place into Microsoft Word tables and RevMan 5.0.

Detailed extraction of qualitative data took place into NVivo 9 software, using text conversion of pdf files to import the whole paper. Coding was then applied to methodological and other potential sources of variation (such as population, intervention and settings), as well as results, to allow data to be assembled in the most appropriate way [46-48].

Study authors were contacted for additional or missing information, where needed.

### Validity assessment

Appropriate validity assessment criteria were developed based on published checklists [44,49]. Data from grey literature were assessed using the same criteria. Two reviewers assessed each study for validity using piloted forms. Disagreements were resolved by discussion and a third reviewer if necessary. No papers were excluded on the basis of the validity assessment.

Each validity assessment form required the reviewer to make an overall assessment of internal validity and of relevance, based on the completed checklists. These were: 1–3 for internal validity (where 1 = good internal validity and 3 = poor internal validity), and a-c for relevance (where a = highly relevant and c = not very relevant).

The quality of cost-effectiveness papers were assessed using a modified version of the Drummond et al. checklist [50]. For papers reporting economic evaluations alongside clinical trials, this was supplemented with reference to the Good Practice Guidance produced by the ISPOR Task Force on Economic Evaluations alongside Clinical Trials [51]. For papers reporting cost-effectiveness models, the checklist was supplemented with reference to the checklist proposed by Drummond et al. [50] and the Good Practice Guidance [51].

### Data synthesis

Quantitative data was synthesised by two reviewers. Where data were suitable for statistical meta-analysis, studies were combined using a fixed effect model to give relative risks with 95% CIs for binary outcomes and weighted or standardised mean differences with 95% CIs for continuous outcomes. Statistical heterogeneity was examined using the χ^2^ and I^2^ statistics, with a χ^2^ p-value of >0.1 or an I^2^ value of >50% indicating statistical heterogeneity, in which case, reasons for the heterogeneity would be investigated, and a random effects model would be used.

A thematic synthesis of qualitative studies was undertaken to combine evidence [46] using QSR NVIVO software to manage the data and ensure a transparent process.

A mixed method systematic review design similar to that used by the EPPI-Centre [46] was then used to combine qualitative and quantitative data. For Review Question 1, studies were grouped according to intervention mode and then type of outcome. For Review Questions 1 and 3, qualitative themes on outcomes for peer deliverers and recipients were mapped to quantitative results grouped by intervention mode and then type of outcome [52].

Due to lack of detail given in the included studies, it was not possible to look at the modifying effects of type of institution, prisoner pathway or gender differences.

For the cost-effectiveness review, data were synthesised through a narrative review with tabulation of results of all included studies.

## Results

**Figure 1:**
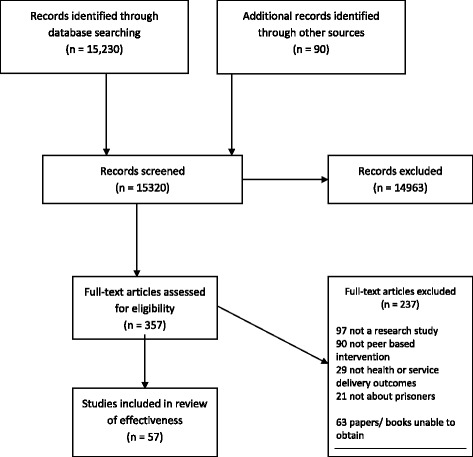
**Study selection process – effectiveness review.**

The effectiveness literature search identified 15,320 potentially relevant papers (Figure 1). 14,963 articles were excluded at the title and abstract screening stage, and 357 articles were obtained and screened in full. 237 papers were subsequently excluded, and we were unable to obtain a further 63 potentially relevant articles, leaving 57 studies included in the review.

**Figure 2:**
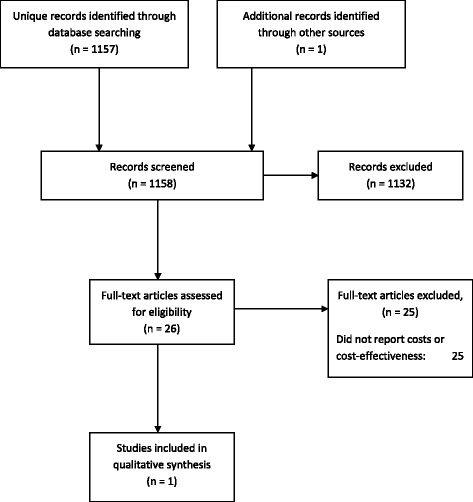
**Study selection process – cost-effectiveness review.**

Searches for the cost or cost-effectiveness analysis of prison-based peer-interventions identified 1158 titles and abstracts (Figure 2). Twenty six full-text papers were retrieved for assessment. From these, one eligible study was identified, 25 studies were excluded on methodological grounds as none reported costs or cost-effectiveness.

A list of excluded studies is available in the full report [41].

The effectiveness review included 57 studies [19,21,23-29,32,36,37,53-98], and one study was included in the review of cost-effectiveness [99,100] (Table 1). Twenty were carried out in the UK (Table 2). Peer education was the most studied intervention mode, followed by peer support (Table 3). Twenty studies looked at HIV/ AIDS/Hepatitis C or other blood borne virus or STI prevention [21,25,27,28,55,60,63,65,66,68,69,75,78,84,85,87,89,93,97,98], 12 at general health and/ or hygiene,(25, 32, 38, 40, 119, 127, 133, 136, 140, 148, 149, 157, 159) eight at general emotional support,(146, 151–156, 161) and seven at prevention of suicide or self-harm. [20,24,32,36,56,61,86] (Table 4)

**Table 1:** **Included Studies**

**Study**	**Country**	**Study design**	**Health topics**	**Nature of intervention/scheme**	**Population/setting**	**Individual outcomes**	**Service, delivery or organisation outcomes**	**Validity** score*
Ashton 2010 [75]	Canada	Qualitative	HIV/AIDS and HCV (& other infectious diseases)	Peer support	“Healing Lodge” – a small (28 bed) minimum/medium security prison for Aboriginal women, incorporating Aboriginal healing practices, meaningfulness and cultural-connection. Most women are serving sentences of 3 years or less.	Strengths of programme listed.	Not reported	3b
						Staff perceptions.		
Betts-Symond 2011 [76]	Ireland	Qualitative	Health, hygiene and cleanliness	Peer education	700 prisoners in Wheatfield prison, Dublin Ireland (medium-high security male prison) and their immediate family members	Personal development and changed outlook of the volunteers; results presented under 6 themes: Environment, behaviours, capabilities, beliefs and values, identity & goals.	Relationship between operational health services and inmate IRC volunteers.	3c
Blanchette 1998 [58]	Canada	Mixed Qualitative& Quantitative	General emotional/ mental health, psychological support and counselling	Peer support	Women resident in one of four small prisons in Canada: Nova Institution; Etablissement Joliette; Grand Valley Institution; Edmonton Institution.	Self-esteem;	Staff and prisoners’ awareness and perceptions of the role and functioning of the PST (surveys);	2b
						Sociometric tests for understanding personal and group dynamics;		
						Perceptions of the prison environment (correctional environment status inventory);		
						Staff and prisoners’ views, feelings and ideas about PST (interviews).		
Boothby 2011 [53]	UK	Qualitative	General health/ support	Peer support	Male prison in the UK.	Insiders perceptions of role and themselves.	Numbers of prison staff	1a
					The scheme supports prisoners who are new to the prison system.	prisoners’ mood; suicide rates		
Boyce 2009 [59]	UK	Mixed	Housing/resettlement	Peer advisors	Serving prisoners in:	skills and self-confidence, work ethic,	Effects on ‘professional’ time.	2a
					3 category B prisons (male), 1 Youth Offending Institution (male)	sense of control over their lives, work experience and qualifications.	Staff concerns: potential for bullying or intimidation and breaches of confidentiality.	
Brooker & Sirdifield 2007 [54]	UK	Mixed Qualitative & Quantitative	Multiple health issues	Health Trainers	Serving prisoners in 4 adult prison, one Young Offenders Institution and one probation setting	Perceptions of tutors of the Health Trainers re. confidence; knowledge of services; communication skills; ability to assess someone’s readiness to change; self-esteem; self-worth.	Perceptions of prison-based trainees re. their role.	1a
						Perceptions of health trainers re. knowledge of health issues and attitude; confidence in sign-posting individuals to services; changing own behaviour.	Perceptions of stakeholders re:	
						Perceptions of health trainer clients; issues discussed; services referred on to.	-workload for prison PE departments	
							-training sessions	
							-Raising risk issues	
							- engagement with health services	
							-Change of focus for the gym	
							-Highlighting a lack of health services in some areas	
							-Raising staff awareness of health issues and/ or services available	
Bryan 2006 [60]	USA	Quantitative Pre-test post-test design (one group only).	HIV prevention	Peer education	196 serving prisoners in maximum and minimum security prisons. 90% male, mean age 30.4y.	Knowledge; Perceived risk; Condom attitudes; Condom norms; Condom self-efficacy; Condom intentions; Attitudes for not sharing needles; Norms for not sharing needles; Self-efficacy for not sharing needles; Intentions to not share needles; Peer education attitudes; Peer education norms; Peer education self-efficacy; Peer education intentions; Peer education behaviour.	Not reported	2b
Chen 2006 [29]	Israel	Quantitative Pre & Post	General emotional/ mental health, psychological support and counselling	Peer counselling	93 male repeat offenders in three prisons in Israel. (Two maximum security and one minimum security).	Sense of coherence; Meaning in life;	Not reported	2b
					Mean age 36 years (SD = 6.35).	Anxiety; Depression; Hostility:		
Cichowlas & Chen 2010 [77]	USA	Qualitative	General health/ support	Prison hospice volunteers	Ill/dying prisoners at Dixon Hospice in Illinois	Perceptions of peer deliverers	Not reported	3c
Collica 2007 [78]	USA	Quantitative & Qualitative	HIV/AIDS and HCV (& other infectious diseases)	Peer education	All prisoners in USA were covered by the survey.	Facilities were asked to report on:	Not reported	3c
						1. Number of HIV positive inmates in their custody;		
						2. If they mandated HIV testing;		
						3. If they provided prison-based peer programming on HIV.		
						If answer to Q3 was YES:		
						Extent of HIV peer education, and other services.		
						If answer to Q3 was NO:		
						How HIV education was provided and why inmate peers were not used.		
Collica 2010 [55]	USA	Qualitative	HIV/AIDS and HCV (& other infectious diseases)	Peer education	Aimed at women in prison with HIV/AIDS.	Role of peers	Not reported	1b
					One maximum and one medium security prison for women			
Correctional Service of Canada 2009 [79]	Canada	*Quantitative & Qualitative*	General emotional/ mental health, psychological support and counselling	Peer Support	Women prisoners “in distress”	From interviews: predominant mental health issues of women prisoners; how these are addressed in training sessions; benefits to trained peer counsellors	Trust between staff and prisoners	3c quant/3b qual
						From survey: whether prisoners value the PST; reasons for asking to see a peer counsellor; benefits to service recipients; helpfulness of peer counsellors; recommendations for improvements	Staff becoming part of peer support team	
							Recommendations for improvements.	
Daigle 2007 [24]	Canada	Not applicable	Suicide/Self harm	Peer support	Canadian prisons (no further details reported).	Not reported	Concerns about recruitment, security and responsibility	N/A
Davies 1994 [32]	UK	Qualitative	Suicide/Self harm	Listeners	HMP Swansea (adult prison)	Attempted suicide rate.	staff time.	2b
						use of the strip cell or care room.	Prison atmosphere.	
						Listeners’ perceptions (benefits to Listeners)		
Delveaux & Blanchette 2000 [80]	Canada	*Quantitative & Qualitative*	General emotional/ mental health, psychological support and counselling	Peer support	Small women’s prison.Women prisoners, all serving sentences of two or more years and classified as minimum or medium security.	Self esteem; Sociometric tests for understanding personal and group dynamics; Perceptions of the prison environment (correctional environment status inventory)	Staff and prisoners’ awareness and perceptions of the role and functioning of the PST (surveys)	3c
						Staff and prisoners’ views, feelings and ideas about PST (interviews).		
Dhaliwal & Harrower 2009 [61]	UK	Qualitative	Suicide/Self harm	Listeners	Vulnerable or distressed prisoners, or those at risk of suicide.	Listeners’ own experiences, the impact on them as individuals, skills and/or benefits acquired.	Presents findings in relation to what the prison service can do to support the scheme.	2b
Dolan 2004 [27]	Russia	Quantitative: pre and post	HIV/AIDS and HCV (& other infectious diseases)	Peer education	Male colony for drug-dependent prisoners in Siberia. Mean age 24 (range 18–30), 63% first time in prison, mean years served 1.2 (SD 0.7), 66% imprisoned for drug related offence.	Whether seen the program booklet?	Access to bleach and condoms	3c
						Whether participated in peer training education?		
						Demographic characteristics; Knowledge of HIV transmission; STI and BBVI status; Drug use; Sexual activity; Tattooing; Access to bleach and condoms.		
Eamon 2012 [81]	Canada	*Quantitative & Qualitative*	General emotional/mental health, psychological support and counselling	Peer Support	Edmonton Institution for Women population = 65	Satisfaction with/ performance of PST;	Suggestions for improvement to number of sessions	3b
						Hours per week of support provided by PST members; Time to response to inmate calls for peer response; Level of trust in PST members; Suggestions for improvement; Improving relationships.		
Edgar 2011 [23]	UK	*Quantitative & Qualitative:*	Multiple health issues	Peer support/ Listeners	Not stated	Various, including Listeners and other peer roles.	Diverting workload away from staff.	2b
Farrin (undated) [82]	Australia	Review	Multiple health issues	Peer support	*At-risk prisoner* in 8 state prisons	Changes in responsibility, accountability and self-esteem (Syed & Blanchette 2000)	Reports the results from Devilly et al., 2003 on changing attitudes and behaviours; Offender preference	3c
Foster 2011 [56]	UK	Qualitative	Suicide/Self harm	Listeners	Adult category-B local male prison. Operational capacity 1103	Effect on Listeners’ personal development; Self-esteem; well-being; relationships.	Prison environment, burden on prison staff and health care professionals.	1a
						Numbers of potential suicides and incidents of self harm.		
Goldstein 2009 [83]	USA	Quantitative	Mental health/Substance abuse	Peer mentoring	2 correctional facilities. Incarcerated women with current or history of behavioural issues and/ or substance abuse.	Adherence to outpatient psychiatric treatment, including medication management; Medication compliance, sobriety & symptom reduction; Re-offending; Abstinence in the use of alcohol or illegal drugs or misuse of prescription drugs; Employment or enrolment in an educational program or completion of the application process for disability benefits; Secure treatment, transitional housing or a permanent place to live.	Nor reported	3c
					Age range: 19 to 59 y (mean = 35 y). 15 out of the 32 participants had 5 or more prior incarcerations.			
Grinstead 1997 [84]	USA	Quantitative: RCT	HIV	Peer education	Male inmates at large (n = approx. 5600) medium-security state prison. . 45% had history of injection drug use, more than 75% of these reported having shared equipment.	HIV Knowledge; Preference for teacher;	Not reported	3b
						Condom use intention; Bleach use intention; HIV antibody use intention;		
						Interested in taking test now.		
Grinstead 1999 [25]	USA	Quantitative. RCT	HIV prevention	Peer education	Large state prison for men. Mean age 35y, spent more than 9y of life in prison. 90% had just completed a sentence of less than 5y and <10% were imprisoned for the first time.	Risky behaviour at follow up:	Not reported	3c
						used a condom the first time they had sex since release; used drugs since release; injected drugs since release; shared needles		
Hall & Gabor 2004 [36]	Canada	Mixed quantitative and qualitative.	Suicide prevention	Listeners	Medium security prison with capacity 585. Inmates have committed serious crimes.	personal growth, knowledge of suicide, self-esteem, communication skills, and sense of purpose; support; general program operation; impact of training; personal development	Findings are reported related to program implementation	3c
					modal age category 18-29y, followed by 30-39y. Length of sentence ranged from 2 years to life.			
Hoover & Jurgens 2009 [85]	Moldova	Qualitative	HIV/AIDS and HCV (& other infectious diseases)	Peer outreach	7 prisons (6male prisons and 1 female prisons)	Not reported	Decline in HIV cases	3c
Hunter & Boyce 2009 [57]	UK	Qualitative	Housing/resettlement	Peer advisors	Prisoners requiring housing advice in 5 prisons in SE England (Three Category B prisons (male), one young offender institution (male) and one female open prison.)	social interaction with others; experience and qualifications to assist post-release; self-confidence.	Views of prisoners and staff re. staff workload and prisoners’ use of their time in prison.	1a
Jacobson & Edgar (undated) [62]	UK	Qualitative	General health/ support	Peer support	New arrivals at HMP Edinburgh	Effects on prisoners	Use of staff time	2c
Junker 2005 [86]	USA	Quantitative	Suicide/Self harm	Peer Observers	Those prisoners judged to be suicidal	Not reported.	Number of hours individuals spent on suicide watch post-IOP compared to pre-IOP (i.e. using staff for observations):	3b
Levenson & Farrant 2002 [19]	UK	Quantitative & Qualitative	Multiple health issues	Peer support/ Listeners.	Not stated	Perceptions of role ( peer supporters)	Not reported	3b quant/2b qual
						Self-esteem.		
						finding accommodation and small amounts of money after release		
Martin 2008 [63]	USA	Quantitative.	HIV/ HCV prevention	Peer education	3 sites: Delaware, Kentucky and Virginia.	The only outcome reported is condom use during sex.	Not reported	2b
		RCT.						
					N = 343. Mean age 34y. 86% male.			
Maull 1991 [64]	USA	Study design unclear	General health/support	Prison hospice volunteers	Ill prisoners at U.S. Medical Centre for Federal Prisoners in Springfield, Missouri	Effects on volunteers;	Retention/attrition of volunteers	2b
						Effects on prisoners		
McGowan 2006 [87]	USA	Qualitative	HIV counselling	Peer education	Male prisoners in state prisons in California, Mississippi, Rhode Island and Wisconsin. aged between 18 and 29y, incarcerated for at least 90 days, classified as minimum or medium security level, scheduled for release within 14 to 60 days.	Effect son HIV testing: mandatory testing at intake, voluntary testing at medical intake, and voluntary testing during a peer health orientation class.	Not reported	3c
Mentor 2 work [73]	UK	Study design unclear	Unclear	Peer mentoring	Prisoners with mental health problems at HMP Liverpool.	Self-esteem, confidence and motivation; Self-worth; Communication skills, reasoning and reflection skills; Mental health and treatment.	Numbers of volunteers and prisoners being mentored; effects after release.	3c
Munoz-Plaza 2005 [65]	USA	Qualitative	HIV/ AIDS and HCV (& other infectious diseases)	Peer education	A state correctional facility in California. Drug treatment program is located on a medium security prison yard that houses male inmates. age range 20–50 years	Not reported	Not reported	2b
O’Hagan 2011 [88]	UK	Quantitative	Literacy	Peer education	Serving Young Offenderss at 5 YOIs	Literacy:	Not reported	3c
						Impact on learners;		
						Impact on mentors		
Peek 2011 [89]	UK	Quantitative	Infectious disease prevention: screening and vaccination.	Peer education	Male prisoners at HMP High Down Category B male local prison.	Hep B and Hep C awareness and vaccination uptake.	signposting to healthcare,	3c
						Chlamydia awareness and screening.	Effects on nurses utilising their time in the prison.	
							Effects on barriers between nursing staff and prisoners.	
							Prison atmosphere.	
							Changing role/perception of prisoners.	
Penn State Erie 2001 [90]	USA	Mixed methods	Parenting	Peer education	Fathers in prison. State Correctional Institute at Albion (SCI Albion), in Erie county. A medium-security institution for men	contact with children per month/year;	Staff awareness and perceptions of programme	3c
						Anger & Frustration; Knowledge about their child/children; Parental Locus of Control; ICAN Fathering Profile; Total Parenting score		
						Father’s Questionnaire: knowledge,		
						attitudes, skills, and behaviors.		
Player & Martin 1996 [91]	UK	Study design unclear	Addictions/substance abuse	Peer counselling	Prisoners with addictions at HMP Downview	drug use; prisoner behaviour	Not reported	3c
Richman 2004 [92]	UK	Quantitative	General emotional/ mental health, psychological support and counselling	Listeners	HMP Manchester	Change in demeanour.	Effects on staff – peer worker relationship.	3b
						Expected effects on release from prison (on Listeners)		
Ross 2006 [66]	USA	Quantitative Pre & Post	HIV/ AIDS and HCV (& other infectious diseases)	Peer Education	36 Texas State prison units. Peer educators and students were predominantly male, aged 34–43 y.	HIV–related knowledge; self–assessed educator skills among peer educators; Diffusion of HIV–related knowledge;	impact of the peer education program on HIV testing at participating units	2b
						HIV–testing behavior and intentions		
Schinkel & Whyte 2012 [67]	UK	Qualitative	Housing/resettlement	Peer mentoring	Based in Glasgow – prisons not stated. Prisoners serving sentences of between three months and four years. Service offered to eligible prisoners who are returning to Glasgow, Renfrewshire and North Lanarkshire.	Effects on prisoners	Staff perceptions of life coaches’ need for support.	2b
Schlapman & Cass 2000 [93]	USA	Quantitative – pre and post	HIV prevention	Peer education	Incarcerated adolescents in North central Indiana juvenile facility.	AIDS knowledge & self reported sexual behaviours.	Not reported	3c
Scott 2004 [68]	USA	Mixed quantitative (pre and post) and qualitative)	HIV prevention	Peer education	Prisoners at 5 Texas prison facilities. A diversity of facilities was selected (small and large, short and long term, male and female prisoners)	HIV related knowledge, attitudes and beliefs among peer educators and students.	Factors affecting implementation, maintenance and overall impact of the program from the perspective of program coordinators, wardens and peer educators.	2b quant/2c qual
Sifunda 2008 [69,101]	South Africa	Quantitative Pre & Post	HIV/ AIDS and HCV (& other infectious diseases)	Peer education	4 medium-sized correctional facilities (male) in South Africa. Number housed comparable in size to UK prison..N = 263. Mean age 27 y (range 17–55). Mean period of incarceration = 2 years (range 6 m – 17 y).65% were first time offenders.	Knowledge and beliefs; Attitudes; Sexual communication, social norms about gender relations and sexual violence;	Not reported	2c
						Self-efficacy; Intentions		
Sirdifield 2006 [70]	UK	Qualitative	General health/ support	Health Trainer	All prisoners	Changes in Health Trainers’ attitudes and health behaviour.	demands placed on prison staff and health services as a result of the intervention.	2b
						Recognising stress in other prisoners.		
Snow 2002 [37]	UK	Quantitative	Suicide/ self harm	Listeners	5 prisons having a Samaritan supported Listener scheme. All prisons were local type establishments and chosen because of the comparatively high rate of suicide.	Perceived benefit from using the scheme:	Not reported	2b
						Approachability of listeners		
						Availability of listeners		
						Use of listener scheme in the future.		
						Reasons for not using the scheme		
						Ways to improve the scheme		
Stewart 2011 [94]	UK	Quantitative & Qualitative	General health/ support	Peer support	3 UK prisons.	Effects on prisoner-carers	communication between staff and prisoners. Training and supervision issues.	3c
					Originally for older prisoners but to include those with learning disabilities, mental health problems and prisoners with physical and sensory disabilities.		Contribution to the health and social care services within the gaol.	
Syed & Blanchette 2000 [95]	Canada	Quantitative & Qualitative	General emotional/mental health, psychological support and counselling	Peer Support	Small women’s prison, n = 78 at time of study. All were serving sentences of minimum 2 years and were rated at ‘minimum’ or ‘medium’ security levels.	Self esteem; Sociometric tests for understanding personal and group dynamics; Perceptions of the prison environment (correctional environment status inventory);	Staff and prisoners’ awareness and perceptions of the role and functioning of the PST (surveys);	3b quant/ 1c qual
					Survey respondents, average age 34.5y (sd = 9.07, range 21–58). Average sentence length 4.39y (range 2 to 15y). Average time spent at Grand Valley = 9 months (SD = 0.62, range = 2 weeks to 2 years).	Staff and prisoners’ views, feelings and ideas about PST (interviews).		
Syed & Blanchette 2000 [96]	Canada	Quantitative & Qualitative	General emotional/ mental health, psychological support and counselling	Peer Support	women’s prison in Canada. N = 56 at time of study. All were serving sentences of minimum 2 years and were rated at ‘minimum’ or ‘medium’ security levels.	Self esteem; Sociometric tests for understanding personal and group dynamics; Perceptions of the prison environment (correctional environment status inventory);	Staff and prisoners’ awareness and perceptions of the role and functioning of the PST (surveys)	3b quant/ 2b qual
					All women, average age 35.1y (SD = 11.3, range = 21 to 62). Average sentence length 4.7 years (range 2y to life). Mean time served at Joliette = 13.3 m (range 2 m to 2.5y).	Staff and prisoners’ views, feelings and ideas about PST (interviews).		
Taylor 1994 [97]	Australia	Quantitative and Qualitative: Pre-post	HIV prevention	Peer education	New South Wales Correctional Centres. 90% of inmates had been in other correctional centres.	Knowledge; attitudes	Awareness of the peer education scheme.	3b
The Learning Ladder Ltd. (undated) [74]	UK	Qualitative.	Mentoring for education/to improve qualifications	Peer mentoring	HM Young Offenders Institution Reading – a small prison holding prisoners between the ages of 18 and 21y.	self-esteem; confidence; attitude to offending behaviour.	Success of scheme.	3c
Vaz 1996 [28]	Mozambique	Quantitative, pre-post	HIV/ STD prevention	Peer education	Largest prison in Mozambique (1900 prisoners incarcerated at time of study). 300 inmates sentenced to 1 year or longer, selected on entry. Mean age 26y.	knowledge around HIV/AIDS ; relationship between knowledge of HIV/AIDS and educational attainment of participants.	Not reported	3b
Walrath 2001 [71]	USA	Quantitative Pre & Post	Violence	Peer training.	Medium all-male security corrections facility in Maryland, USA, housing inmates serving sentences of 3 months or longer.	Anger; Self esteem; Optimism; Locus of Control; Behaviour	Not reported	2b
					Age range: 18 to 51 y, mean age 30 y. Average sentence 20y, ranging from less than 1 year to life.			
Wright & Bronstein 2007 [72,102] *2 papers*	USA	Mixed Qualitative & Quantitative	General health/ support	Prison hospice volunteers	Dying prisoners in 14 prison hospices in the USA	Not reported	Impact of having a hospice (& implicitly, using prisoner volunteers) on prison environment & climate.	2c
Zack 2001 [21]	USA	Quantitative	HIV/AIDS and HCV (& other infectious diseases)	Peer education	Medium-security prison housing approximately 6000 men who stay at the prison for an average of less than two years. Men arriving at and leaving the prison, and women visitors.	Intentions to use condoms and be tested for HIV; Knowledge; HIV/AIDS testing; behaviour	Resistance from staff	3b
							Institutional lockdowns	
		RCT						
Zucker 2006 [98]	USA	Quantitative. One-group pretest - posttest.	Hepatitis C prevention	Peer education	Massachusetts county jail . 25 men who spoke and wrote in English.	Changes in self-reported behaviour, knowledge, relationship with teacher .	Not reported	3c

NOTE: Validity score: 1 = good internal validity, 2 = moderate internal validityand 3 = poor internal validity; a = highly relevant, b = of some relevance, and c = not very relevant.

**Table 2:** **Number of included studies by Country**

**Country**	**Number of studies**
USA	20
UK	20
Canada	9
Australia	2
Ireland	1
Israel	1
Moldova	1
Russia	1
Mozambique	1
South Africa	1

**Table 3:** **Number of included studies by intervention mode**

**Intervention mode**	**Number of studies**
Peer education	21
Peer support	14
Listeners	6
Peer mentoring	4
Prison hospice volunteers	3
Peer advisors	2
Health trainers	2
Peer counselling	2
Peer outreach	1
Peer observers	1
Peer training	1

**Table 4:** **Number of included studies by health topic**

**Health topic**	**Number of studies**
HIV/AIDS/HCV/BBV prevention	20
General health, hygiene	12
Emotional support	8
Suicide/self harm prevention	7
Employment/housing post release	4
Mental health/substance abuse	2
Improving educational skills	2
Parenting	1
Violence reduction	1

Overall, the internal validity of included studies was quite poor, with only five studies judged to be of good quality [53-57], 18 of moderate quality [23,29,32,58-72] and 32 poor quality [19,21,25,27,28,36,73-98]. Five were judged to be highly relevant [53,54,56,57,59], with 27 of some relevance [19,21,23,28,29,32,55,58,60,61,63-68,70,71,75,79,81,84,86,92,95-97] and 22 not very relevant [25,27,36,62,69,72-74,76-78,80,82,83,85,87-91,93,98].

The main issues affecting internal validity were small sample size, lack of comparators and/or lack of adjustment for potential confounding factors, poor reporting of study methodology and poor reporting of results, limiting meta-analysis of quantitative studies, or meta-ethnography of qualitative studies. Only two studies defined “peer”.

A typology of interventions was developed with working definitions for the major intervention modes (Table 5).

**Table 5:** **Types of peer interventions**

**Type of peer intervention**	**Working definition**
**Peer education**	Peer education involves the teaching and communication of health information, values and behaviours between individuals who are of equal social status, or share similar characteristics, or have common experiences [103,104]. Peer education has been widely applied in the prison setting, particularly in relation to HIV prevention and risk reduction. Peer educators typically undertake formal training to equip them with the knowledge and skills to undertake the role.
**Peer support**	Peer support is the support provided and received by those who share similar attributes or types of experience. Peer support can be an informal process between individuals and/or can be provided through formalised interventions where peer supporters seek to promote health and/or build people’s resilience to different stressors [104]. There is a range of different peer support interventions reported in the prison literature. In the UK, the Listeners scheme is a specific peer support intervention focused on prevention of suicide and self-harm.
**Prison hospice volunteers**	Prison hospice volunteers provide companionship, practical assistance and social support to terminally ill patients. They may be involved in a range of activities as requested by patients including letter writing, reading, accompanying patients to religious services and other parts of prison and sometimes maintain a bedside vigil with dying patients [102].
**Mentoring**	Mentoring describes the development of a relationship between two individuals where the mentee is able to learn from the mentor, model positive behaviour and gain experience, knowledge or skills [105,106]. Peer mentors, as defined by Finnegan et al., have a similar background or experiences to their mentee ([106]:6). There are a number of peer mentoring schemes in UK prisons focused on education and training, such as The Learning Ladder [74], and on resettlement and prevention of reoffending.
**Health trainers**	Health trainers are lay public health workers who use a client-centred approach to support individuals around health behaviour change and/or to signpost them to other services, some of which are also free at the point of delivery (Health Trainers England). Prison health trainers receive the standardised training on health promotion, healthy lifestyles and mental health, but adapted for the prison setting and client group.

### Review Question 1: What are the effects of peer-based interventions on prisoner health?

Fifty-one studies were relevant to review question 1 [19,21,25,27-29,32,36,53-64,66-77,79-84,86-98], eighteen had a quantitative design [21,25,27-29,60,63,66,69,71,83,84,86,88,89,92,93,98], three of these were RCTs [25,63,84]. Fourteen studies had a qualitative design [32,53,55-57,61,62,67,70,74-77,87,107] and 15 were mixed methods [19,36,54,58,59,68,72,79-81,90,94-97]. Four studies had an unclear design [64,73,82,91]. Seventeen were UK studies [19,32,53,56,57,59,61,62,67,73,74,88,89,91,92,94] and 17 were from the USA [21,25,55,60,63,64,66,68,71,77,83,84,86,87,90,93,98]. The predominant intervention type was peer education (19 studies) [21,25,27-29,55,60,63,66,68,69,76,84,88-90,93,97,98].

Findings are presented in Table 6.

**Table 6:** **Review Question 1 findings**

	**Intervention type:**						
	**Peer Education**	**Peer support**	**Listeners**	**Prison hospice volunteers**	**Peer mentoring**	**Health trainers**	**Other**
***Knowledge***	Ten studies [66,68,69,84,88,90,93,97,98]	Two qualitative studies showed reported increases in knowledge [58,80]. In one of these studies, a number of respondents noted that knowledge acquired from the training was applicable to improving relationships with their children, partners and others in the community [58].	Enhanced skills as a result of being a peer deliverer, like listening and communication, was mentioned by two studies [56,61] and there was indication of prisoners feeling able to put these skills into practice on release from the institution [61].			Two qualitative studies showed increased knowledge on a variety of topics, including: drugs, sexual health, nutrition, alcohol and mental health issues [54].	
	Statistically significantly higher proportion of correct answers to 22/ 43 questions asked in peer education vs control group. RR 0.43 (95% CI: 0.33, 0.56, 1 study n = 949) to 3.06 (95% CI: 1.91, 4.91, 1 study, n = 200).					Improvements were seen in the mean knowledge scores in all areas in one study [54], but it was not possible to ascertain whether these improvements were statistically significant.	
	Knowledge scores: mean difference 0.46 (95% CI: 0.36, 0.56, 2 studies, n = 2494, I^2^ = 94%).					Both health trainers and Health Trainer tutors reported that Health Trainers had developed effective communication and listening skills as well as fostering attributes essential for team working and future employment after release from prison [54].	
	Other evidence: peer educators improved their own knowledge [55,68,76]. and [69] information was diffused to those outside the prison, such as family members and children.						
	In the study on literacy [88], > 90% of learners agreed that their reading and communication skills had improved.						
***Intentions***	Four studies [66,69,84,93]		In one study [37] 61% of those surveyed said they could talk to a Listener about anything that was worrying them. 74% had no problems contacting a Listener when they had requested help.				
	One RCT [84] reported improvements in: interest in taking HIV test for the first time (RR 1.49, 95% CI: 1.12, 1.97);		57% of users thought they would seek the help of a Listener if they faced a similar problem in the future.				
	interest in taking HIV test now (RR 1.82, 95% CI: 1.33, 2.49); condom use intention (RR 1.15, 95% C I: 1.08, 1.22);						
	intention to never use condoms (RR 0.59, 95% CI: 0.48, 0.72).						
	No improvement in intention to use bleach with drug injecting equipment (RR 1.06, 95% CI: 0.97, 1.16).						
	No improvement [67] in intention to take a HIV test (RR 1.24, 95 CI: 0.75, 2.05) and a negative effect on peer educators’ intentions (RR 0.62, 95% CI: 0.41, 0.95).						
	A study in South Africa [69] did not show any evidence of a commitment to change their behaviours, X^2^(10, N = 69) = 10.934, p = .36.						
***Attitudes/ Beliefs***	Four studies [68,69,97,98]	One study [91] showed that a drug treatment intervention that included the support of trained prison counsellors caused changes in prisoners’ reported attitudes to drugs and alcohol. This translated to a self-reported reduction in drug and alcohol use. The one-to-one sessions with trained peer counsellors was regarded as the most “helpful aspect” of the recovery process.				Attitudinal change, often as a result of increased knowledge, was seen primarily in the area of smoking and diet [54,70]..	
	No changes in one study [68]; in another [97], improvements seen in agreement with all three statements:					In one study [54], more than 50% of health trainers stated that their attitude had changed in the areas of: healthy eating/ diet; sexual health issues; smoking cessation; exercise; mental health issues. 75% of HTs stated that they would like to get a job as a HT when they are released from prison	
	“HIV positive inmates should be separated” (RR 2.55, 95% CI: 1.94, 3.33);						
	“I feel safe in the same wing as an inmate who is HIV positive” (RR 0.74, 95% CI: 0.68, 0.84);						
	“I know enough to protect myself from catching HIV/AIDS” (RR 0.54, 95% C: 0.50, 0.59).						
***Behaviour***	Eleven studies [21,25,27,60,63,66,68,69,87,89,90,98]		In one study [92], 64% of 22 prisoners claimed that friends and family had noticed a difference in their demeanour, finding them more relaxed, responsible, optimistic, able to speak more and more able to listen. 73% agreed that their new responsibilities would allow them to ‘adjust better’ on release, and 55% agreed that the ‘prison authorities’ appreciated their work. 77% said there was a difference in how immediate staff interacted with them: being trusted more, staff talking more to them, staff being grateful for the work they do. 86% said that fellow prisoners behaved differently towards them.		In one study [83] At 3 months, 38/44 participants (86%) were receiving outpatient psychiatric services and 40/44 (91%) successfully managing their medications.	Health trainers reported eating more fruit and vegetables and one health trainer had given up smoking [54,70]	**Peer training:** One study [71] reported a statistically significantly reduced rate of confrontation post-intervention at 0.432 (CI: 0.319, 0.583, p < 0.0005).
	Positive effects seen:				At 6 months, 36/44 participants (82%) were medication compliant, and 35/44 (80%) demonstrated symptom reduction. 12/44 (27%) had not maintained sobriety at 6 month time point. 17/22 (77%) participants released for at least 12 months had not been rearrested. 16/22 participants who had been released for at least 12 months (73%) were abstinent in use of alcohol or illegal drugs or misuse of prescription drugs.		
	Not using a condom at first intercourse after release from prison (RR 0.73, 95% CI: 0.61, 0.88, 2 studies, n = 400);						
	injecting drugs after release from prison (RR 0.66, 95% CI: 0.53, 0.82, 2 studies, n = 400);						
	injected in past 4 weeks (RR 0.11, 95% CI: 0.01, 0.85, 1 study, n = 241);						
	sharing injection equipment after release from prison (RR 0.33, 95% CI: 0.20, 0.54, 2 studies, n = 400);						
	peer educators never having had an HIV test (RR 0.31, 95% CI: 0.12, 0.78, 1 study, n = 847).						
	In one Russian study [27] the prevalence of tattooing in prison significantly decreased (42% vs 19%, p = 0.03) and of those who were tattooed the proportion using a new needle increased from 23% to 50%.						
	Where behaviour was measured on a scale [60,69,98], positive effects were seen in all three studies.						
	HIV tests in prison [87] was associated with having attended a HIV prevention programme in prison (OR = 2.81, 95% CI: 1.09, 7.24).						
	Chlamydia screening in the under-25 s rose from 13 to 83 in a 6 month period after beginning a peer education intervention, similarly hepatitis C screening increased from 9 to 46, and numbers were also increased for HIV screening and hepatitis B vaccinations [89].						
	In a study on parenting skills [90] statistically significant improvements in self-reported father/ child contact were seen (mean difference 41.3, 95% CI: 6.47, 76.13).						
***Confidence***	One study [69] reported no significant differences.	No statistically significant effect of the peer intervention in three studies [58,80,95] (WMD 1.51, 95% CI: −0.84, 3.86, 3 studies, n = 83, I^2^ = 81%).	Trained individuals reported that they were ‘giving something back’, doing something constructive with their time in prison and being of benefit to the system; this consequently had an effect on individuals’ self-esteem, self-worth and confidence [19,23,32,36,56,61].	Volunteers experience increases in self-esteem and self-worth as a result of the service they provide to others [72,102]. Evidence also suggests prisoners gain an enhanced sense of compassion for other people [72,102] and being prison hospice volunteers allows individuals ‘to give something back’ [77].		Health trainers seemed most confident in signposting to exercise, smoking cessation and drugs services and least confident in signposting to self-harm, immunisation and dental services [54].	**Peer training:** One study [71] reported s mall but statistically significant negative effects of the intervention on self- esteem (MD −2.15, 95% CI: −4.20, −0.10), measured with the Rosenberg self-esteem scale, and optimism (MD 1.30, 95% CI: −0.83, 3.43), measured with the life orientation text.
		Qualitative evidence suggested improvements in the peer deliverers’ self-esteem, self-worth and confidence as a result of the role [53,58,79-81,96].The sense of being trusted by the prison authorities to counsel and support prisoners in distress was reported to enable peer deliverers to regain their self-respect [23,79].The notion that peers became more empowered consequentially was alluded to [58,79,80,95,96].				Qualitative research [54] found that training as a health trainer had been a huge boost to prisoners’ confidence, self-esteem and self-worth, reported by key staff. There was also evidence of health trainers bolstering other prisoners’ reported self-esteem and confidence through listening and supporting individuals [54].	**Peer outreach:** Qualitative evidence suggested that peer volunteers felt that their role was worthwhile and that they were making a difference to the health of the prison population [85].
							**Peer advisers: Two studies reported** increased self-esteem and self-confidence, coupled with peer deliverers reporting that they were building a work ethic and a sense of control over their lives [57,59]. The role was perceived by the volunteers to be worthwhile and purposeful as well as enabling social interaction with others and offering ‘structure’ to the prison day [57]
***Mental health***	No effect on anger or frustration in the parenting skills study [92], either immediately post-intervention (MD 0.20, 95% CI: −1.42, 1.82) or at longer follow-up (MD 1.40, −0.03, 2.83).	Peer support was reported to have helped prisoners either practically, emotionally, or both [58] and in one study it was demonstrated that this type of intervention could be particularly beneficial for prisoners during the early part of their sentence [62]. Those who had used peer support reported using it as an avenue to vent and to overcome feelings of anxiety, loneliness, depression and self-injury [58,79,96] and there were indications that this may be potentially beneficial in preventing suicides in prison [53].	Three studies [32,36,56] reported an impact in reducing depression and anxiety in distressed prisoners and improving their mental state. There is anecdotal evidence that suicide and self-harm is reduced as a result of the support offered by peers acting in this role. A fourth study [37] found 44% of users of the Listener scheme reported that they always felt better after confiding in a Listener, while 52% felt better at least 'sometimes'. 84% said they had always found the experience helpful.	In one study, prison volunteers described life enrichment, growth, and coming to terms with their own mortality as a result of their involvement [64]. Moreover, the recipients of one of the programmes suggested how the volunteers had supported them and enabled them to overcome states of depression [64].			**Peer training:** One study [71] found no statistically significant effect of the intervention on anger (mean difference −4.01, 95% CI: −9.40, 1.38), measured with the anger expression scale.
			Four studies [32,56,61,92] related the emotional burden of listening to other prisoners’ problems and issues. Discussions relating to suicidal intentions and other distressing topics could be particularly burdensome for peer deliverers to manage. There were also reports of peer deliverers experiencing ‘burnout’ and mental exhaustion as a result of the demands placed on their time by other prisoners [56,92]				**Peer support and counseling:** One study [29] looked at the effects of peer support (Narcotics Anonymous meetings) and counselling (12 step programme), compared to peer support alone (NA meetings only) on mental health, namely coherence, meaning in life, anxiety, depression and hostility. Improvements with the combined interventions were seen in all outcomes: coherence (mean difference −0.31, 95% CI: −0.48, −0.14), meaning in life (MD −0.42, 95% CI: −0.65, −0.19), anxiety (MD −0.42, 95% CI: −0.66, −0.18 ), depression (MD −0.35, 95% CI: −0.52, −0.18 ), hostility (MD −0.11, 95% CI: −0.18, −0.04).
***Preference***	In an American HIV RCT [84], 68% preferred to be taught by an inmate with HIV versus 11% who preferred a HIV/ AIDS educator.						
***Additional themes***	Qualitative evidence suggested that peer deliverers found the experience personally rewarding, giving their time in prison meaning and purpose [55,68]. In one study, this included improved listening and communication skills as a result of their participation [90]. Other [55research suggested that being a peer educator also enabled the difficulties of prison life to be off-set through the supportive network of other trained peer educators.	No statistically significant effect was seen on prisoners’ perceptions of the prison environment in the pooled results of 3 studies [58,80,95]			16/22 (73%) participants released for at least 12 months were employed, enrolled in an educational program or had completed the application process for disability benefits.	**Prisoner outcomes:** Issues most likely to be discussed with health trainers were reported in one study [54] to be exercise, weight and healthy eating.	**Peer observers:** One controlled study [86] found a statistically significant decrease (t(71.55) = 2.14, p = 0.036) in the mean number of hours on watch following the implementation of the Inmate Observer Programme.
		One study [79] found that 81% of 35 respondents valued the existence of the Peer Support Team. Another study [81] reported that inmates were very satisfied with the quality of the information delivered by PST members. Expectations of the PST were also well met.			18/22 (82%) participants who had been released for at least 12 months had secured treatment, transitional housing or a permanent place to live.	**Onward referrals:** Health trainers in one study [54] were most likely to refer clients to gym staff or healthcare staff. Referrals were also made to Counselling, Assessment, Referral, Advice, and Throughcare services (CARATS), counsellor, dentist and optician.	
		Staff reported that PST members were effective in handling crisis interventions, providing services to inmates and serving as role models.					
		In one study [81] PST members estimated that they provided support to others of 3–5 hours per week on average.					
		In several studies [23,58,79,80,96], there was indication of peer deliverers gaining better self-awareness and perspective on their life as well developing the skills to deal with their own health and offending issues. There was limited information on the impact that the role would have on future re-offending. Only in one study [23] was it suggested that the experiences of being a peer support worker would be beneficial in reducing the likelihood of re-offending.					
		The demands placed on peer support worker/counsellors by other prisoners gave individuals a sense of purpose in prison [23,53,94] and this was beneficial for combatting boredom while serving the prison sentence [23,53].However, there were indications that the role could be challenging and onerous and the burden of care of supporting many prisoners could be problematic [53].					

#### Peer education

**Figure 3:**
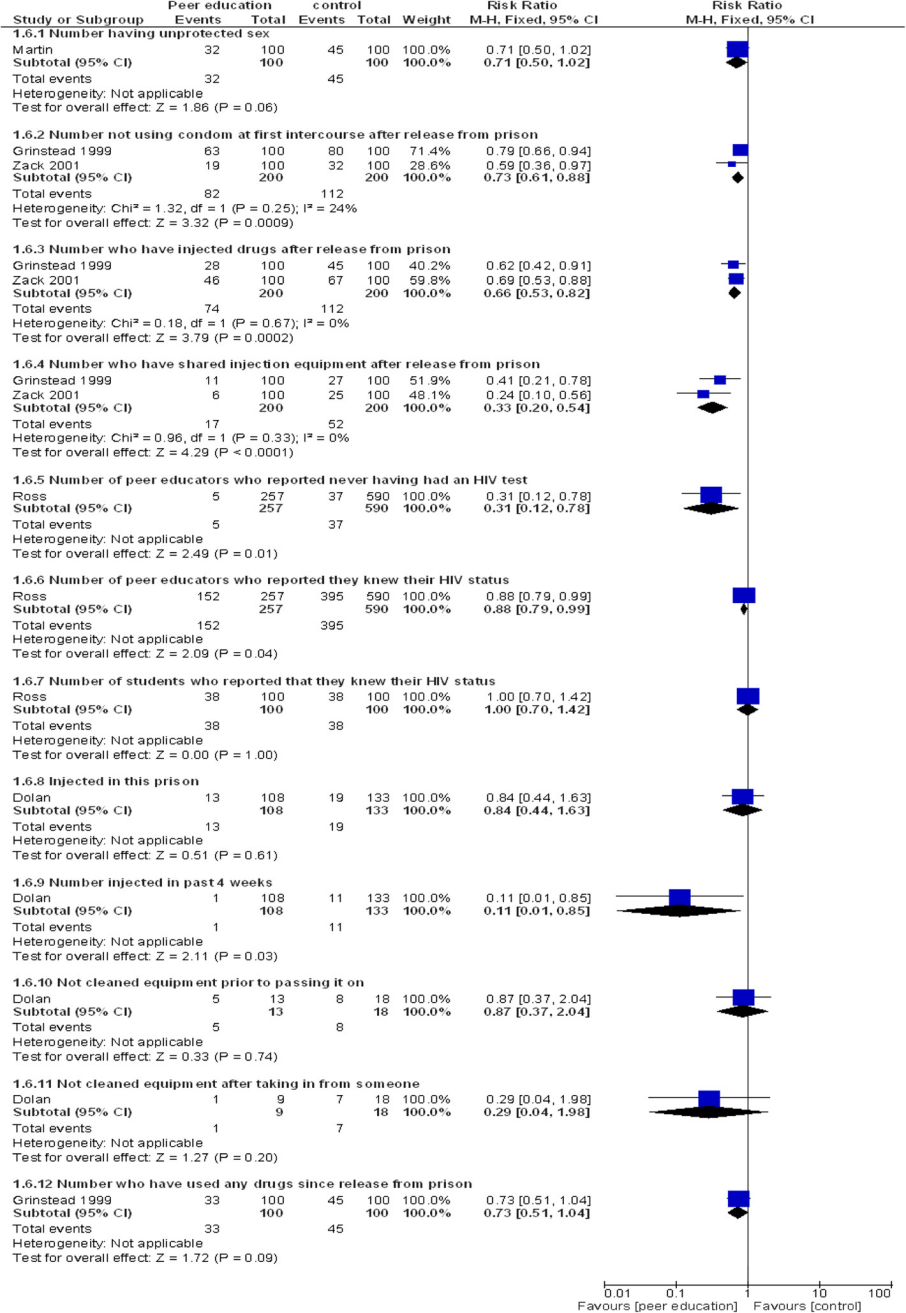
**Effects of peer education on behaviour (binary outcomes).**

Ten included studies [28,66,68,69,84,88,90,93,97,98] reported the effects of peer education on prisoner knowledge. There was no standard outcome measure used. Statistically significant improvements favouring peer education were seen in the number of correct answers to 22 of the 43 questions asked, while negative effects of peer education were seen in the answers to one of the 43 questions asked. The responses to the remaining 20 questions showed no evidence of effect of the intervention. Risk ratios ranged from 0.43 (95% CI: 0.33, 0.56, 1 study n = 949) - in favour of peer education to 3.06 (95% CI: 1.91, 4.91, 1 study, n = 200) - against peer education (Figure 3). Qualitative evidence suggested that peer educators improved their own knowledge of health issues as a result of their training [55,68,76].

Findings were equivocal for the effects of peer education on behaviour change intentions and health beliefs. Consistent evidence indicated that peer education reduced risky behaviours: not using a condom at first intercourse after release from prison (pooled RR 0.73, 95% CI: 0.61, 0.88, 2 studies, n = 400); injecting drugs after release from prison (pooled RR 0.66, 95% CI: 0.53, 0.82, 2 studies, n = 400); injected in past 4 weeks (RR 0.11, 95% CI: 0.01, 0.85, 1 study, n = 241); sharing injection equipment after release from prison (pooled RR 0.33, 95% CI: 0.20, 0.54, 2 studies, n = 400); peer educators never having had an HIV test (RR 0.31, 95% CI: 0.12, 0.78, 1 study, n = 847) (see Figure 2). Weak evidence indicated an association between peer health education programmes and uptake of screening/HIV testing in prisons [87,89].

#### Peer support

Six included studies reported the effects of peer support interventions on prisoners [58,79-81,95,96]. These all reported on the Canadian Peer Support Team (PST) program and used similar evaluation designs and outcome measures. The PST Program is a model that has been developed and delivered across a number of Canadian prisons. It is specifically targeted at women prisoners and is based on a holistic, women-centred approach to health care that aims to be culturally sensitive and to develop the women’s autonomy and self-esteem. Three studies used the Rosenberg self-esteem scale to measure prisoners’ self-esteem [58,80,95] and found no statistically significant effect (pooled WMD 1.51, 95% CI: −0.84, 3.86, 3 studies, n = 83), although there was substantial heterogeneity (I^2^ = 81%). Strong qualitative evidence related to improvements in the peer deliverers’ self-esteem, self-worth and confidence as a result of the role [53,58,79-81,96]. The sense of being trusted by the prison authorities was reported to enable peer deliverers to regain their self-respect [23,79]. The notion that peers became more empowered consequentially was alluded to [58,79,80,95,96]. Peer support was reported to have helped prisoners either practically, emotionally, or both [58] and could be particularly beneficial for prisoners during the early part of their sentence [62]. In several studies [23,58,79,80,96], peer deliverers gained better self-awareness and perspective on their life as well as developing the skills to deal with their own health and offending issues. One study [23] suggested that the experience of being a peer support worker could reduce the likelihood of re-offending.

The demands placed on peer support workers/counsellors by other prisoners gave individuals a sense of purpose in prison [23,53,94] and this was beneficial for combatting boredom while serving the prison sentence [23,53]. However, there were indications that the role could be challenging and onerous and the burden of care of supporting many prisoners could be problematic [53].

#### Listeners

Strong qualitative evidence supported individual health gains for those trained as Listeners or befrienders. Trained individuals reported that they were ‘giving something back’, doing something constructive with their time in prison and being of benefit to the system; this consequently had an effect on individuals’ self-esteem, self-worth and confidence [19,23,32,36,56,61]. Moreover, enhanced skills as a result of being a peer deliverer, like listening and communication, were mentioned by two studies [56,61] and there were indications of prisoners feeling able to put these skills into practice on release from the institution [61].

There were some negative health effects reported [32,56,61,92] and these related to the emotional burden of listening to other prisoners’ problems and issues.

Two interventions studied in the UK, health trainers and peer mentors, focused on changing behaviours. One study provided weak evidence that mentoring had positive effects on health behaviours, treatment adherence, drug taking and re-offending [83]. Two studies provided moderate evidence that becoming a health trainer positively affected knowledge, attitudinal and behaviour change, self-esteem and development of transferable skills [54,70]. There was little evidence of effects on health trainers’ clients; however limited evidence showed that health trainers discussed a range of lifestyle issues with clients and referred them to other services [54,70].

Twenty-one predominantly qualitative studies [19,23,29,32,36,53-59,61,64,68,72,79-81,96,102] indicated that being a peer worker was associated with positive effects on mental health and its determinants. These findings were consistent across a number of different models including peer education, peer support, Listeners, prison hospice volunteers, health trainers, and Peer Advisors. Skill development, including transferable employment skills, was also mentioned in relation to Peer Advisors [57,59] and health trainers [54]. Negative effects for peer workers related to experiencing a burden of care, particularly in roles involving emotional support [32,56,61,92].

### Review Question 3: What is the effectiveness of peer delivery compared to professional delivery?

Very few studies compared peer-led to professionally-led interventions. Three of four quantitative studies were about peer education for HIV prevention [21,63,84], two of which were RCTs [63,84]. Consistent evidence from these studies indicated that peer educators are as effective as professional educators in HIV prevention. The fourth was a study of peer observers for suicide watch [86].

Consistent evidence from ten qualitative studies [23,54,56-59,67,80,95,96] indicated that peer delivery was preferred to professional, with cross cutting themes including peer deliverers demonstrating empathy due to lived experiences, being non-judgemental, being trusted by prisoners and offering more time than staff. Prisoners felt more at ease talking to fellow prisoners and also found them more accessible.

### Review Question 4: What is the cost-effectiveness of peer based interventions in prisons?

Only one study met the inclusion criteria [99,100], focusing on costs rather than health outcomes, and the programme aim was poorly described. The study showed management cost savings in prisons in the short term through the use of a Therapeutic Community (TC) programme, albeit these were a small part of the overall costs. Their findings suggest that TC activities or the existence of the TC environment may help to reduce or control prison management costs.

## Discussion

Overall, current evidence is strongest in terms of evaluating effects on peer deliverers, rather than recipients of peer interventions. There is strong evidence that being a peer worker is associated with positive effects on mental health and its determinants, and this is consistent across a number of peer intervention models. Peer education interventions are effective in reducing risky behaviours, and peer support services are acceptable to prisoners and have a positive effect on recipients. There is some evidence that peer educators are as effective as professional educators for HIV prevention outcomes, and strong evidence that peer delivery is preferred to professional delivery. The finding of reduced risk of HIV transmission was in part reflective of the fact that it was the outcome that was most commonly evaluated. Therefore the absence of evidence for other health outcomes should not be misinterpreted as evidence of absence of the effectiveness of peer education for addressing health conditions other than HIV. Research into cost-effectiveness is sparse, with little economic evaluation even into interventions with evidence of effectiveness.

The 58 included studies represent the best available evidence, although their methodological quality was limited. Most did not report an underpinning theoretical model and only two defined ‘peer’, which leads the reader to make their own assumptions about whether peer deliverers and recipients within prisons are a homogeneous group. The dominance of positive findings in the quantitative data suggests publication bias. Clinical heterogeneity in outcomes and interventions precluded meta-analysis of most outcomes. Studies of interventions delivered by non-professionals, and studies of prison health, are not well indexed in electronic databases and early pilot searches returned impractically large numbers of hits. A more specific search strategy was developed, but this may have lost some sensitivity and therefore some relevant studies may have been missed. The effects of peer interventions on reoffending and other non-health outcomes (such as employment) are not represented in this review, nor are the effects of non-prisoner volunteers on prisoner health, effects of peer interventions in the probation service, or staff-to-staff peer interventions, although there is a body of literature on each of these. 63 studies were unobtainable: 17 were books and another substantial proportion were PhD theses or newspaper or magazine articles. Not all would have met inclusion criteria.

A previous review in this area highlighted a lack of evidence-based literature on the efficacy of prison-based peer-led programmes [22], but this review only searched one database, and included only peer education interventions. Nevertheless, their conclusions concurred with ours, showing prison-based peer education programmes as well tolerated, effective and possibly more cost-effective than professionally led programmes. A 2011 systematic review of peer education for health promotion in prisons [31] searched fewer databases than our review, including only ten studies, and concluded, as does our review, that peer education is effective in reducing risk of HIV transmission.

This is the first systematic review of all the evidence on effectiveness and cost-effectiveness of peer interventions in prisons, a topic that is now of considerable interest to the Department of Health for England and Wales and NHS England. Given that the WHO consensus statement on mental health promotion in prisons argues that activities should be available to help offenders make best use of their time inside, and that the Prison Reform Trust estimates that only 20% of prisoners will be employed whilst inside (in industrial workshops for example), there is a need to provide meaningful occupation for offenders. Being a peer worker could provide such meaningful occupation [108], moreover peer-based interventions can be considered a valuable mechanism to maintain or improve health and wellbeing in the prison setting. A recent study of peer based interventions in mental health services found that peer workers were able to engage people with services by building relationships based on shared lived experience [109]. The benefits of peer education and support, particularly in those pathways that are concerned with changing behaviour or requiring individual motivation to pursue a healthy lifestyle, have also been seen in other areas such as managing substance misuse and addiction [110,111], and managing long-term conditions (for example, the Expert Patient Programme [112]).

This study has highlighted research gaps and ways in which the evidence base for peer-based interventions in prison settings could be strengthened. This work supports the Health and Justice function in Public Health England who have called for evidence-based guidelines and advice on all aspects of public health in prisons, including health promotion and public health [113]. It is vital that to further inform the evidence base, future studies need to be methodologically robust, sufficiently broad to capture outcomes for different stakeholder groups and assess costs and benefits both within and outside the prison system. Research is needed to explore the impact across the criminal justice system in line with the Department of Health’s focus on offender health and understandings of the wider determinants of health in this vulnerable group.

There is also a pressing need for implementation and economic evaluation of a prison based peer educator initiative.

## Conclusions

Peer-based interventions can be considered a valuable mechanism to maintain or improve health and wellbeing in the prison setting, with positive effects seen on knowledge and behaviour of peer deliverers and recipients. Peer education is less used in prisons in England and Wales than in the USA, perhaps reflecting more general trends in health promotion; however, the finding that peer education can increase knowledge and reduce risky health behaviours, particularly in relation to HIV prevention, suggests that consideration should be given to whether a peer education component should be introduced into other health behaviour change interventions.

### Transparency statement

All authors had full access to all of the data and can take responsibility for the integrity of the data and the accuracy of the data analysis. Dr Bagnall affirms that the manuscript is an honest, accurate, and transparent account of the study being reported; that no important aspects of the study have been omitted; and that any discrepancies from the study as planned have been explained.

### Ethics approval statement

The study received approval from the National Offender Management Service (NOMS) National Research Committee (Ref: 165–11) and the research team agreed to conduct the study in compliance with the Terms and Conditions set out by the National Research Committee. The study did not require ethical approval through NRES. Study documentation was reviewed through the Faculty of Health and Social Sciences Research Ethics Committee, Leeds Beckett University.

### Data sharing

Full search strategies and lists of included and excluded studies are available from the corresponding author at a.bagnall@leedsbeckett.ac.uk.

## Abbreviations

BBV: Blood Borne Viruses
HCV: Hepatits C Virus
CI: Confidence Interval
DARE: Database of Abstracts of Reviews of Effects
DoPHER: Database of Promoting Health Evidence Reviews
EPPI: Evidence for Policy and Practice Information
HIV: Human Immunodeficiency Virus
HMP: Her Majesty’s Prison
HSDR: Health Services and Delivery Research
NHS: National Health Service
NHSEED: National Health Service Economic Evaluation Database
NIHR: National Institute for Health Research
NOMS: National Offender Management Service
OHRN: Offender Health Research Network
PCT: Primary Care Trust
POA: Prison Officers Association
PORSCH: Prison and Offender Research in Social Care and Health
RePEc: Research Papers in Economics
WHO: World Health Organisation
YOI: Young Offenders’ Institution
